# Spatial Assessment of Potentially Toxic Elements (PTE) Concentration in *Agaricus bisporus* Mushroom Collected from Local Vegetable Markets of Uttarakhand State, India

**DOI:** 10.3390/jof8050452

**Published:** 2022-04-27

**Authors:** Pankaj Kumar, Vinod Kumar, Ebrahem M. Eid, Arwa A. AL-Huqail, Bashir Adelodun, Sami Abou Fayssal, Madhumita Goala, Ashish Kumar Arya, Archana Bachheti, Željko Andabaka, Kyung Sook Choi, Ivan Širić

**Affiliations:** 1 Agro-Ecology and Pollution Research Laboratory, Department of Zoology and Environmental Science, Gurukula Kangri (Deemed to be University), Haridwar 249404, Uttarakhand, India; rs.pankajkumar@gkv.ac.in (P.K.); drvksorwal@gkv.ac.in (V.K.); 2 Biology Department, College of Science, King Khalid University, Abha 61321, Saudi Arabia; ebrahem.eid@sci.kfs.edu.eg; 3 Botany Department, Faculty of Science, Kafrelsheikh University, Kafr El-Sheikh 33516, Egypt; 4 Department of Biology, College of Science, Princess Nourah bint Abdulrahman University, P.O. Box 84428, Riyadh 11671, Saudi Arabia; aaalhuqail@pnu.edu.sa; 5 Department of Agricultural and Biosystems Engineering, University of Ilorin, PMB 1515, Ilorin 240103, Nigeria; adelodun.b@unilorin.edu.ng; 6 Department of Agricultural Civil Engineering, Kyungpook National University, Daegu 41566, Korea; 7 Department of Agronomy, Faculty of Agronomy, University of Forestry, 10 Kliment Ohridski Blvd, 1797 Sofia, Bulgaria; sami.aboufaycal@st.ul.edu.lb (S.A.F.); ks.choi@knu.ac.kr (K.S.C.); 8 Department of Plant Production, Faculty of Agriculture, Lebanese University, Beirut 1302, Lebanon; 9 Nehru College, Pailapool, Affiliated Assam University, Cachar, Silchar 788098, Assam, India; madhumitagoalap@gmail.com; 10 Department of Environmental Science, Graphic Era (Deemed to be University), Dehradun 248002, Uttarakhand, India; ashishkumararya_20941076.evs@geu.ac.in (A.K.A.); bachheti.archana@gmail.com (A.B.); 11 University of Zagreb, Faculty of Agriculture, Svetosimunska 25, 10000 Zagreb, Croatia; zandabaka@agr.hr

**Keywords:** cluster analysis, health risk, potentially toxic elements, mushroom, spatial analysis, principal component

## Abstract

This study presents a spatial assessment of eight potentially toxic elements (PTE: Cd, Cr, Cu, Fe, Pb, Ni, Mn, and Zn) in white button (*Agaricus bisporus* J.E. Lange) mushroom samples collected from the local vegetable markets of Uttarakhand State, India. Fresh *A. bisporus* samples were collected from thirteen districts and fifteen sampling locations (M1-M15) and analyzed for the concentration of these PTE using atomic absorption spectroscopy (AAS). The results revealed that *A. bisporus* contained all eight selected PTE in all sampling locations. Based on the inverse distance weighted (IDW) interpolation, principal component (PC), and hierarchical cluster (HC) analyses, the areas with a plane geographical distribution showed the highest PTE concentrations in the *A. bisporus* samples as compared to those in hilly areas. Overall, the decreasing order of PTE concentration in *A. bisporus* was recognized as Fe > Zn > Mn > Cr > Cu > Ni > Cd > Pb. The Kruskal–Wallis ANOVA tests displayed a highly significant (*p* < 0.05) difference among the sampling locations. However, the concentration of PTE was below permissible limits, indicating no potential hazard in consuming the *A. bisporus*. Similarly, the health risk assessment studies using the target hazard quotient (THQ) also showed no significant health risk associated with the consumption of *A. bisporus* being sold in the local mushroom markets of Uttarakhand, India. This study is the first report on state-level monitoring of PTE in *A. bisporus* mushrooms, which provides crucial information regarding the monitoring and occurrence of potentially toxic metallic elements.

## 1. Introduction

Around the globe, the consumption of mushrooms is gaining attention due to their unique flavor and high nutritional value compared to meat and vegetables [1]. Their nutritional value is mainly related to the composition of substrates on which they are grown, including the type of added supplements. Therefore, substrates (e.g., composts), including raw and casing materials, play a crucial role in the production, preservation, and marketing of quality mushrooms [2]. The close interrelationship between substrates and mushrooms prompts researchers to investigate possible dangers to human health as a result of mushroom consumption. *Agaricus bisporus* (button mushroom) is considered a strong bio-accumulator of potentially toxic elements (PTE), especially mercury (Hg), lead (Pb), and cadmium (Cd) [3,4]. The mushroom usually grows on composted lignocellulosic materials, which can contain considerable levels of PTE [5]. Accordingly, *A. bisporus* could be a natural “treatment plant” for different agro-industrial, dairy, and domestic wastewaters [6], resulting in reduced hazardous dispersion of PTE in the environment. However, not all button mushrooms that are consumed are a product of human cultivation, especially in developing countries where the local population tends to forage and sell the mushroom in rural markets as their main source of income [7,8]. The PTE concentrations of these wild mushrooms may be very high if they are collected near mines or industrial factories [9].

Potentially toxic elements are non-degradable elements that can accumulate and pose high risks to human health when found in high concentrations in the food chain [10,11,12,13,14]. Although several PTE (e.g., Cu, Fe, Zn, Ni) are essential elements for the human body, when consumed at high concentrations, they can cause fatal neurological disorders, allergic reactions, abnormal hemoglobin content, growth retardation in children, hemolysis, and nephrotic effects [3,15,16]. On this basis, several countries and commissions, such as the USEPA, WHO, and FAO/WHO Codex, have specified maximum limits of PTE in mushrooms intended for human consumption [17,18].

Uttarakhand, one of the 28 states of India, contains 45.44% forest area, making it a plentiful location for several saprophytic and mycorhizal fungal species [19,20,21]. Uttarakhand State has been contributing to increasing mushroom production in India since its establishment in the year 2000. Recent figures found the annual mushroom production in Uttarakhand to be 10236 metric tons [22]. Out of this total, *A. bisporus* is the most commonly produced, and is obtained from three sources: (i) collected from wild forest areas, (ii) commercially grown on farms, and (iii) locally grown by small farmers [23]. In this state, the majority of cultivated mushrooms are sold in the local vegetable markets. For the cultivation of *A. bisporus*, the farmers use agricultural residues of wheat crop (straw). Since it is observed that PTE contents can be transferred to the edible parts of mushrooms through the process of bioaccumulation, it is therefore necessary to monitor their levels in mushrooms sold in the local vegetable markets in Uttarakhand State.

Sinha and co-workers previously investigated the PTE contents and their availability in button mushrooms collected from two markets in other states of India, including Maharashtra [3]. They observed copper (Cu), iron (Fe), zinc (Zn), manganese (Mn), cadmium (Cd), and lead (Pb) contents in *A. bisporus* lower than the safe limits reported by USEPA, Indian Standards, WHO, and FAO [18,24]. However, a large spatial assessment of PTE concentrations in mushrooms collected from Uttarakhand State markets within the districts has not yet been conducted. Therefore, the current study investigated the spatial PTE contents in *A. bisporus* mushrooms collected from fifteen local markets located in different areas of Uttarakhand State, India.

## 2. Materials and Methods

### 2.1. Description of the Study Area and Sample Collection

**Figure 1:**
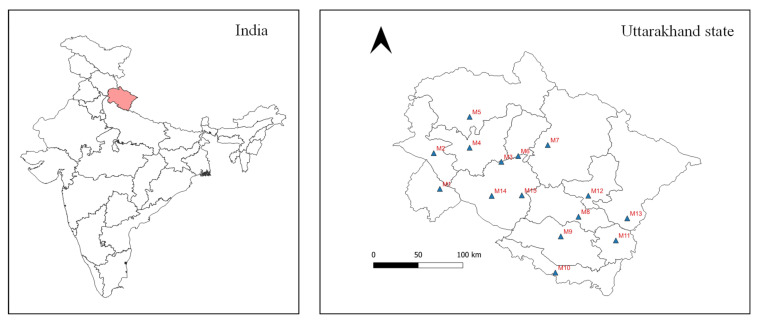
Map view of the study area (▲ sampling sites).

The current study was conducted in the Uttarakhand State of India. Uttarakhand State is one of the 28 states of India with a total area of 53,483 km^2^ and a population of 11.5 million. Over 86% of the total area is covered by mountains while 45.44% is covered by forests. This makes Uttarakhand a rich habitat for many edible and non-edible macrofungi [20,21]. The mushroom samples were collected from fifteen sampling sites (M1 to M15) located in each of the thirteen districts in Uttarakhand State. Figure 1 and Table 1 show the geographical distribution of *A. bisporus* sample collection sites. The sampling locations were divided into two groups, namely hilly and plain areas, based on their elevation. Specifically, the fresh fruiting bodies of *A. bisporus* mushroom were collected from local vegetable markets of the selected locations. The collected mushroom samples (*n* = 3 for each market) were washed thoroughly to remove any adhering dirt or soil particles and dried using blotting paper.

The mushroom samples were collected from November 2021 to January 2022, which is the most suitable period for seasonal *A. bisporus* cultivation by local growers. The samples were immediately transported to the laboratory in a polyurethane foam insulated ice cooler box (11 L, PinnacleThermo, Ahemdabad, Gujrat, India) and oven-dried (KI-181, Khera Instruments Pvt. Ltd., Delhi, India) at 60 °C until the constant weight of biomass was achieved. The samples were converted into a fine powder using a mechanical grinder (HL7576/00 600 W, Philips Amaze Ltd., Solan, India).

### 2.2. Analytical Methods

The concentration of PTE in mushroom samples was analyzed by using atomic absorption spectroscopy (AAS: A-Analyst 800, PerkinElmer, Waltham, USA). For this, 1 g dehydrated mushroom powder was mixed in a di-acid mixture (5 mL HNO_3_ + 2.5 mL HClO_4_) and left overnight for self-digestion (12 h). Further, the sample was adjusted to 50 mL using a 3% HNO_3_ solution followed by heating digestion on a hot plate (150 °C for 1 h) until a 10 mL sample was left. Finally, the digested sample was filtered through Whatman filter paper no. 41 and supernatants were used for PTE quantification (i.e., Cd, Cr, Cu, Fe, Pb, Mn, Ni, and Zn) using AAS. The detection limits of the instrument for Cd, Cr, Cu, Fe, Pb, Mn, Ni, and Zn were 3, 4, 4, 5, 20, 3, 10, and 3 µg/L, respectively [25,26]. Selective hollow cathode lamps of PTE were cast-off at optimum current and operated by following the standard operating procedures (SOPs) recommended by the manufacturer. The slit width of the instrument was adjusted to 0.5 nm and a mixture of air/acetylene gas was used to run the AAS. Calibration curves were prepared using standard solutions (0 as control, 0.5, 1, 5, 10, 50, and 100 mg/L) of PTE. Qualitative assurance of the PTE analysis results was performed based on the maximum recovery percentage (>98%). All analyses were conducted in triplicate.

### 2.3. Data Analysis

The data of PTE concentration were analyzed using principal component analysis (PCA) and hierarchical cluster analysis (HCA) tools. The principal component analysis is a statistical technique widely used to study the relative contribution of participating data groups based on their correlation or covariance coefficients. By using PCA, eigenvectors and variance values are extracted from the matrix, which allows for a comparison of the dominance of selected variables [27]. These values are extracted as two or more groups based on data coverage known as principal components (PCs). A biplot is drawn from the computed covariance matrix, which reflects the coordinates of the original variable and their relation to participating data groups. Due to its high efficiency in estimating spatial and temporal patterns of environmental and agriculture data, PCA has been explored by numerous researchers [4,28]. Similarly, cluster analysis is a useful technique for identifying data groups having the highest or lowest pairwise similarities [29]. Agglomerative nesting (AGNES) is one of the best hierarchical algorithms for developing similarity that utilizes single element clusters for each group. The result is developed as a form of a combined tree and heatmap that represents similarities between all participating groups [30]. Therefore, PCA and cluster analysis were performed in the current study to draw a covariance matrix, biplots, and clustered heatmaps to understand the relationship between sampling locations and their influence on PTE availability in *A. bisporus* mushroom samples collected from the Uttarakhand State. Moreover, the map-based graphical visualization of the PTE data was conducted using the inverse distance weighted (IDW) interpolation method of the geographical information system (GIS) approach [31].

The health risk associated with the consumption of PTE contaminated *A. bisporus* by the consumers of Uttarakhand State, India was computed using the Target Hazard Quotient (THQ) approach. By using THQ, a health risk index (HRI) is developed, which is used to assess the occurrence of health hazards [31,32,33]. An HRI value above 1 indicates a potential health risk in consuming the contaminated mushroom [34]. For this, THQ and HRI values of *A. bisporus* samples collected from selected sites were calculated by using the following equations (Equations (1) and (2)):

THQ = 10^−3^ × (PTE_EE_ × I_EA_ × PTE_CF_ × PTE_C_)/(I_BW_ × PTE_ACP_ × PTE_RD_) (1)

where, PTE_EE_ is the exposure efficiency of PTE (365 days/year), I_EA_ is the exposure age of an individual (70 years), PTE_CF_ is the consumption frequency of PTE (2.2 g/day), PTE_C_ is the PTE concentration in *A. bisporus* sample (fresh weight basis), I_ABW_ is the average body weight of the vegetable consumer (70 kg and 16 kg for adult and child groups), and PTE_ACP_ is the average consumption period of PTE (25,550 days). PTE_RD_ represents the PTE reference doses of Cd, Cu, Cr, Fe, Pb, Mn, Ni, and Zn, namely, 5.0 × 10^−4^, 4.2 × 10^−2^, 3.0 × 10^−3^, 7.0 × 10^−1^, 2.0 × 10^−2^, 1.4 × 10^−2^, 3.5 × 10^−3^, and 3.0 × 10^−1^ mg/kg/day, respectively [34]. Afterward, the HRI of PTE intake was computed [18,35]:

HRI = ∑THQs(2)

### 2.4. Software and Tools

The data were analyzed using Microsoft Excel 2019 (Microsoft Corporation, Redmond, WA, USA), OriginPro 2021b (OriginLab Corporation, Northampton, MA, USA), and QGIS Desktop (3.22.3-Białowieża, Open Source, Gispo Ltd., Helsinki, Finland) software packages. All values presented in the current study were calculated as mean followed by standard deviation (SD).

## 3. Results and Discussion

### 3.1. Concentration of PTE in A. bisporus Mushroom

The results of the PTE concentration in *A. bisporus* mushroom samples collected from different locations (M1–M15) of Uttarakhand State, India are summarized in Table 2. The statistical results of the Kruskal–Wallis (K-W) ANOVA revealed that the mean PTE concentration in *A. bisporus* samples varied significantly (*p* < 0.05) between the selected sampling locations. The concentration of Cd ranged from 0.06 ± 0.01 to 0.09 ± 0.1 mg/kg at all sites, which is very close to the safe limit (0.10 mg/kg) of Indian Standards [3]. The mean concentration of Cr was recorded as 16.21 ± 2.87 mg/kg. Similarly, the mean concentrations of Cu, Fe, Pb, Mn, Ni, and Zn were recorded as 15.07 ± 2.48, 37.37 ± 8.59, 0.05 ± 0.02, 20.00 ± 3.44, 1.05 ± 0.43, and 36.37 ± 3.39 mg/kg, respectively. Based on the location variation, the highest concentration of PTE was found at multiple sites such as Cd (M3, M9, M10, M11), Cr (M1), Cu (M12), Fe (M1, M3), Pb (M1, M2), Mn (M1, M13), Ni (M1, M12), and Zn (M1). In general, the M1 site showed a relatively higher concentration for Cr, Fe, Pb, Mn, Ni, and Zn PTE. The coefficient of variance (CV < 37.37%) also showed a relatively low error rate in state-wise monitoring of PTE in *A. bisporus* mushroom samples. Overall, no sample was found to have any of the selected PTE below the detection limits of the AAS. The presence of PTE in mushrooms is due to their vital role in fungal growth, metabolism, and reproduction. Elements such as Cu, Cr, Fe, Mn, Ni, and Zn are taken up by fungal mycelia as micro or trace nutrients, which further support their effective growth [36]. However, Cd and Pb are not essential nutrients and may harm mushroom growth if present in the substrate at high concentrations. However, these toxic elements may be taken up by mushroom cell walls as a substitute for other elements and to bring chemical equilibrium in the mycelial growth zone [4].

**Figure 2:**
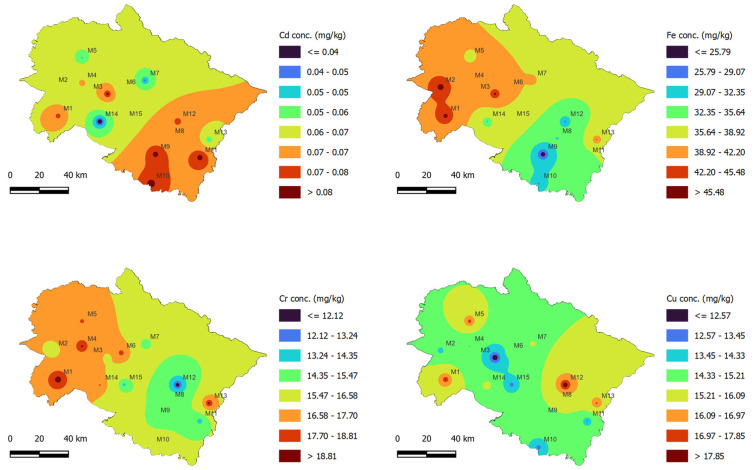
Spatial concentration of Cd, Cr, Cu, and Fe in *A. bisporus* samples collected from different locations (M1–M15) of Uttarakhand state, India.

**Figure 3:**
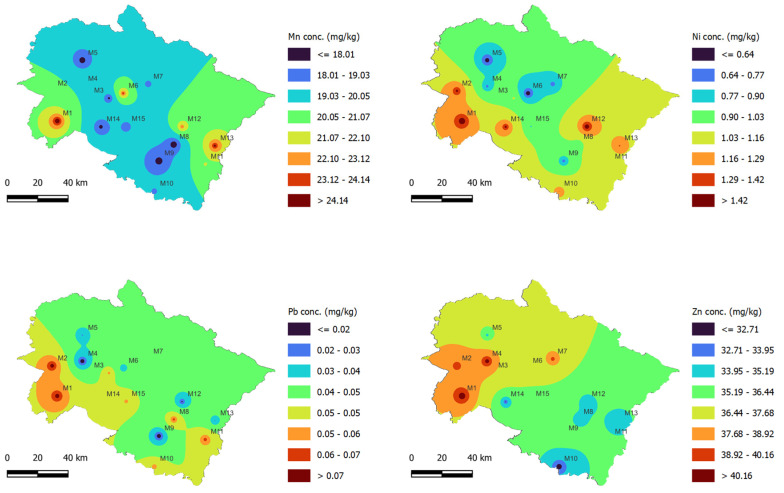
Spatial concentration of Pb, Mn, Ni, and Zn in *A. bisporus* samples collected from different locations (M1–M15) of Uttarakhand state, India.

Based on the contour maps generated using the IDW interpolation tool of QGIS (Figure 2 and Figure 3), the eight districts of Uttarakhand State (M1: Haridwar; M3: Pauri Garhwal; M8: Almora; M9: Nainital; M10: Udham Singh Nagar; M11: Chapawat; M12: Bageshwar, and M13: Pithoragarh) were more affected with Cd contamination, whereas Fe and Cr were highest in the case of the M1 and M2 locations. Similarly, these two locations showed the occurrence of other PTE in the highest concentrations, which might be due to their location in plane areas where the majority of agricultural and industrial activities occur. The mushroom growers use locally available substrates such as wheat straw, wheat bran, animal manures, chemical fertilizers (urea, di-ammonium phosphate, super-phosphate, etc.), and soil-compost mixtures for casing material, which might be the source of the PTE absorbed by the *A. bisporus*.

Based on the altitudinal variation in sampling sites, it is evident that the sites having lower elevation (plane areas) showed higher concentrations of the selected PTE. For instance, M1 is a plane region and its samples exhibited high levels of Cr, Cu, Fe, Pb, Mn, Ni, and Zn contamination. Wheat is the major crop in the M1 region, which can be attributed to the plane terrain and optimal seasonal conditions. The straw obtained after wheat harvesting is mainly used as fodder and for mushroom cultivation. As a result of the extensive utilization of chemical-based fertilizers and pesticides, and irrigation using polluted water sources, the wheat straw may also become contaminated, later affecting the elemental composition and quality of *A. bisporus*. The local mushroom growers of this region utilize wheat straw waste which might be contaminated with this PTE, which later accumulate within the fruiting bodies of *A. bisporus*. Higher elevation sites, by contrast, have limited availability of wheat straw substrate because of the unsuitable climatic conditions (i.e., they are usually cold). However, the sites with higher elevations exhibit good climatic conditions (usually cold) that support the natural occurrence of certain mushroom species including *A. bisporus*. The local markets at higher elevation sites sell *A. bisporus* sourced from both natural and commercial harvesting, which might be a reason behind the lower concentration of some PTE observed for these sites.

Previously, no study has reported a state-level analysis of PTE concentration in *A. bisporus* in the Uttarakhand State, India. However, a study by Singh et al. [37] analyzed the elemental composition of four Ganoderma mushroom species collected from wild forest areas of Uttarakhand State, India. They reported a total of 27 elements that did not exceed the recommended dose reference (RDF) values and were found to be safe for human consumption. Another study by Gaur et al. [38] also investigated the nutritional and elemental composition of seven mushroom species, including *A. bisporus*. Their results revealed that *A. bisporus* had significant PTE contents, including Cu, Cr, Fe, Mn, and Zn as analyzed by AAS. However, they did not report any concentration of Cd, Pb, As, and Hg PTE. Thus, the findings of this study suggest occurrences of several PTE, which did not reach the safe limit but still could pose risks to the consumer’s health.

### 3.2. PCA and Hierarchical Cluster Analysis

**Figure 4:**
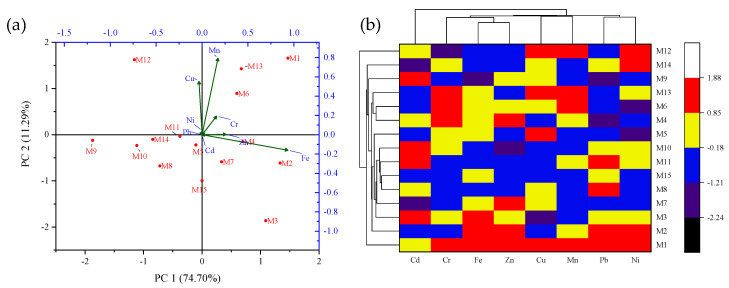
(**a**) PCA biplot and (**b**) clustered heatmap of Potentially Toxic Elements (PTE) concentration in *A. bisporus* samples collected from different locations (M1–M15) of Uttarakhand state, India.

In this study, the interactive effect of sampling location on PTE availability in *A. bisporus* samples was analyzed using the PCA tool. The dimension reduction method of PCA helps in identifying the positive or negative interaction between the input variables [27]. Based on the PCA, the data were transformed into two different principal components (PC1 and PC2). The extracted components, namely, PC1 and PC2, had eigenvalues of 83.47 and 12.61 with the variance of 74.70% and 11.29%, respectively. These PCs were helpful in deriving the interactive effects of input locations and PTE availability in *A. bisporus* samples through the vector lengths given in Figure 4a. Moreover, the data given in Table 3 also shows the dominating PCs of PTE based on their actual vector lengths. The results indicate that Fe concentration was highest at M2, whilst Zn was highest at M1. The concentration of Cr and Mn was also highest at M1 with a positive interaction. On the other hand, Cu, Ni, and Pb exhibited negative interaction with sampling locations, where the highest Cu was observed at M12, and the highest Pb and Ni at the M1 site. Besides this, Cd showed the highest concentration at multiple sites (M3, M9, M10, and M11), indicating a potential health risk posed by this PTE at these locations.

Similarly, the hierarchical cluster analysis (HCA) is a descriptive classification method widely used in identifying the data objects or groups having the highest similarities or dissimilarities [39]. In this study, the similarities among locations were determined based on the closeness of available heavy metal concentration in *A. bisporus* mushroom samples. By this method, a heat map-based clustered diagram (Figure 4b) was produced to understand identifiable data groups. Based on the nearest neighboring method of Euclidean clustering, the minimum and maximum distance identified were 1.77 and 3.96, respectively. Further, the highest similarities were shown by Pb-Ni, Cu-Mn, and Fe-Zn, while two other PTE (Cd and Cr) appeared in quite different individual clusters.

In previous studies, PCA has been a widely used and accepted tool for big data analysis, particularly for PTE in mushroom species. In a study by Buruleanu et al. [40], heavy metal concentrations were determined in the mushroom samples (regional, wild, and cultivated) collected from different locations in Romania. They implemented the PCA tool to assess the interaction between dominating PTE through the Varimax rotation method and found that K, Mg, Cd, and Cr showed the highest positive interaction. Moreover, the findings of this study were in line with those reported by Širić et al. [41], in which they analyzed PTE in 10 saprophytic mushroom species collected from Croatia. The cluster analysis showed the highest similarities between edible mushroom species belonging to the same phenotypic groups. Similarly, Bosiacki et al. [42] investigated PTE levels in wild *A. bisporus* mushroom samples collected from Poland. The HCA analysis helped in identifying similar regions having the highest heavy metal levels in *A. bisporus* samples. Therefore, PCA and HCA were helpful to understand the interactive effects of the availability of PTE in *A. bisporus* mushroom samples collected from the Uttarakhand state of India.

### 3.3. Health Risk Assessment of A. bisporus Mushroom

With an increasing rate of edible mushroom consumption, there has been a need for adequate monitoring of associated heavy metal levels. However, the toxicity of PTE varies largely based on the type and amount consumed. For this reason, toxicity studies using the target hazard quotient (THQ) health risk index (HRI) provide a better insight into the possible health hazard posed to an individual as well as the combined intake. In the present study, THQ and HRI indices were used to obtain critical index values for adult and child human groups. The results showed that the child human group was more susceptible to all eight selected PTE compared to the adult group. As given in Table 4, THQ values vary largely with changes in the sampling location. Notably, the combined HRI values were below 1, indicating no possible health hazard associated with the consumption of *A. bisporus*. More specifically, the highest HRI values were observed at the M1 (Haridwar) site, which might be due to the occurrence of a large number of industrial units (pharmaceutical, electroplating, agro-industrial, papermaking, textile, distillery, etc.) releasing toxic wastes into the environment. Apart from that, the M1 is a plane region with extensive agricultural activities, meaning high use of chemical fertilizers and pesticides [43], so these higher levels could also be a result of bioaccumulation of PTE from agricultural wastes to edible parts of mushrooms. Overall, the decreasing order of HRI in the study area was identified as M1 > M13 > M6 > M4 > M14 > M5 > M10 > M2 > M3 > M8 > M9 > M11 > M7 > M15 > M12. The uptake of PTE by *A. bisporus* depends on several factors such as their bioavailable concentrations in the composted substrate, casing soil, and irrigation water [5]. Moreover, substrate pH and organic matter also play an important role in constructing the substrate–fungal network, which facilitates the migration of PTE to the upper edible parts of the mushroom. The most prevalent PTE, including Cd, Cr, Cu, Pb, Ni, and Zn may pose health risks to the consumer if their intake amount and exposure duration are high. Lately harvested mushrooms may also show a high PTE concentration, as time plays an important role in creating equilibrium between mushrooms and the substrate used to grow them [8].

A study by Igbiri et al. [44] investigated the THQ and HR of PTE in edible mushroom species in the Niger Delta, Nigeria. They observed that the maximum HR values were identified for Ni heavy metal, indicating serious health risks for the consumers. Similarly, Kumar et al. [5] also performed HRI studies for the uptake of PTE by *A. bisporus* cultivated on compost loaded with industrial wastewater. The values of THQ and HRI were below the specified health hazard level. Moreover, Karataş [45] also analyzed the contents of PTE in cultivated oyster mushrooms and performed THQ studies. The results revealed that the contents of six elements (Ca, Mg, Na, Zn, Cd, and Cr) were within permissible limits, indicating the edibility of the cultivated mushroom. Thus, the results of the above-mentioned studies are in agreement with the findings of the current study, thereby suggesting the importance of THQ and HRI indices in determining the health risks associated with the consumption of PTE-containing mushrooms.

## 4. Conclusions

This study analyzed the spatial variations in the concentrations of potentially toxic elements (PTE) in *A. bisporus* mushroom samples collected from different locations in Uttarakhand State, India. The findings of this study reveal that *A. bisporus* samples showed varying contents and concentration levels of eight PTE (Cd, Cr, Cu, Fe, Pb, Mn, Ni, and Zn). The mushroom samples collected from plane regions showed high concentrations of PTE compared to hilly regions. The PCA and HCA tools were useful in identifying the dominance and similarity characteristics of heavy metal availability. The contents of PTE in *A. bisporus* mushroom did not exceed the safe limits, while the health risk index exhibited no potential health hazard associated with their consumption. Thus, this study suggests the *A. bisporus* mushroom being sold in the local vegetable markets were safe for human consumption. Also, this study points out the healthiness and suitability of button mushrooms sold in Uttarakhand State vegetable markets. Further studies on monitoring of other PTE (Hg, As, Co, etc.) in *A. bisporus*, as well as other commercially sold mushroom species, are highly recommended.

## Figures and Tables

**Table 1:** Description of different locations (M1–M15) selected for *A. bisporus* collection in Uttarakhand state, India.

Site Code	Site Name (Vegetable Market)	District	Longitude	Latitude	Elevation (m)
M1	Jwalapur Sabji Mandi	Haridwar	78.10285	29.91545	281
M2	Dehradun Sabji Mandi	Dehradun	78.03421	30.31900	659
M3	Pauri Garhwal Sabji Mandi	Pauri Garhwal	78.79454	30.22143	626
M4	Tehri Garhwal Sabji Mandi	Tehri Garhwal	78.43813	30.38010	1050
M5	Uttarkashi Bus Stand	Uttarkashi	78.43798	30.72878	1141
M6	Rudraprayag Bridge Market	Rudraprayag	78.98489	30.28644	723
M7	Chamoli Gopeshwar Market	Chamoli	79.31739	30.41037	1474
M8	Almora Chandni Chowk	Almora	79.66361	29.60164	1602
M9	Nainital Bus Stand	Nainital	79.46428	29.37957	1936
M10	Udham Singh Nagar Rudrapur	Udham Singh Nagar	79.40320	28.97121	215
M11	Champawat Naad Bora	Champawat	80.08405	29.33380	1691
M12	Bageshwar Saryu Bridge	Bageshwar	79.77404	29.83861	877
M13	Pithoragarh Bus Stand	Pithoragarh	80.21207	29.58334	1503
M14	Lansdown Trishakti Chowk	Pauri Garhwal	78.68650	29.83720	1687
M15	Bironkhal	Pauri Garhwal	79.02541	29.84294	1545

**Table 2:** Concentration (Mean ± SD) of PTE in *A. bisporus* samples collected from different locations (M1–M15) of Uttarakhand state, India.

Site Code	Potentially Toxic Elements (PTE) Concentration (mg/kg)							
	Cd	Cr	Cu	Fe	Pb	Mn	Ni	Zn
M1	0.08 ± 0.01	20.35 ± 0.16	18.26 ± 0.08	48.28 ± 0.62	0.08 ± 0.01	26.35 ± 0.15	1.65 ± 0.10	42.34 ± 1.52
M2	0.06 ± 0.01	15.19 ± 0.28	13.69 ± 0.56	49.35 ± 0.45	0.08 ± 0.01	20.84 ± 0.37	1.51 ± 0.23	40.07 ± 0.80
M3	0.09 ± 0.01	16.02 ± 0.42	10.36 ± 1.30	48.16 ± 0.28	0.06 ± 0.02	17.18 ± 0.40	1.07 ± 0.09	37.20 ± 2.43
M4	0.07 ± 0.01	19.46 ± 0.80	14.21 ± 0.96	42.03 ± 1.34	0.01 ± 0.03	20.07 ± 0.24	0.68 ± 0.06	41.93 ± 1.30
M5	0.05 ± 0.01	18.08 ± 1.07	17.82 ± 0.25	37.28 ± 2.70	0.03 ± 0.02	16.66 ± 0.16	0.47 ± 0.07	34.45 ± 0.65
M6	0.06 ± 0.02	19.06 ± 0.71	14.67 ± 0.30	41.73 ± 1.08	0.03 ± 0.01	24.50 ± 0.70	0.39 ± 0.05	36.85 ± 1.05
M7	0.04 ± 0.03	14.38 ± 1.02	15.44 ± 0.46	40.26 ± 0.55	0.04 ± 0.02	18.15 ± 1.30	0.66 ± 0.12	40.10 ± 0.30
M8	0.07 ± 0.01	15.10 ± 0.45	15.35 ± 0.27	31.82 ± 0.38	0.07 ± 0.01	15.94 ± 0.83	0.90 ± 0.16	34.42 ± 2.73
M9	0.09 ± 0.01	14.72 ± 0.30	14.84 ± 0.02	20.10 ± 3.91	0.01 ± 0.01	16.49 ± 0.32	0.65 ± 0.10	36.00 ± 0.50
M10	0.09 ± 0.01	16.05 ± 0.21	12.73 ± 0.15	28.13 ± 0.56	0.06 ± 0.01	18.76 ± 0.60	1.30 ± 0.24	31.08 ± 0.78
M11	0.09 ± 0.01	13.29 ± 0.59	13.08 ± 0.80	34.19 ± 0.10	0.07 ± 0.01	21.30 ± 1.30	1.10 ± 0.08	35.19 ± 1.35
M12	0.08 ± 0.02	10.23 ± 0.28	19.70 ± 0.11	27.32 ± 0.43	0.02 ± 0.01	23.42 ± 0.45	1.66 ± 0.19	33.87 ± 0.82
M13	0.05 ± 0.01	19.75 ± 0.14	17.36 ± 1.09	43.12 ± 0.22	0.03 ± 0.02	25.10 ± 0.20	1.32 ± 0.14	34.03 ± 0.42
M14	0.02 ± 0.01	18.03 ± 0.53	16.02 ± 0.26	30.54 ± 0.70	0.05 ± 0.01	17.05 ± 0.36	1.54 ± 0.08	32.30 ± 0.36
M15	0.06 ± 0.01	13.45 ± 0.42	12.55 ± 0.08	38.27 ± 0.86	0.06 ± 0.02	18.16 ± 0.30	0.87 ± 0.12	35.74 ± 0.10
Mean ± SD	0.07 ± 0.02	16.21 ± 2.87	15.07 ± 2.48	37.37 ± 8.59	0.05 ± 0.02	20.00 ± 3.44	1.05 ± 0.43	36.37 ± 3.39
CV (%)	31.40	17.68	16.48	23.00	51.05	17.20	40.97	9.32
K-W	0.047	0.001	0.002	0.003	0.042	0.009	0.025	0.019
SL	0.10	20.00	40.00	425.00	0.20	30.00	1.50	50.00
Reference	[3]	[3]	[3]	[18]	[18]	[18]	[3]	[3]

CV: coefficient of variance; SL: safe limits; K-W: Kruskal–Wallis *p*-value.

**Table 3:** PCA matrix results for dominance of Potentially Toxic Elements (PTE) in *A. bisporus* samples collected from different locations (M1-M15) of Uttarakhand state, India.

Potentially Toxic Elements (PTE)	Principal Components	
	PC1	PC2
Variance (%)	74.70	11.29
Eigenvalue	83.47	12.61
Cd	**−0.01**	−0.02
Cr	0.15	**0.19**
Cu	−0.03	**0.55**
Fe	**0.93**	−0.16
Pb	**0.01**	−0.01
Mn	0.17	**0.79**
Ni	0.03	**0.04**
Zn	**0.26**	0.01

Bold values indicate dominating axis for specific PTE.

**Table 4:** Target Hazard Quotient (TQH) and Health Risk Index (HRI) results of PTE in the *A. bisporus* samples collected from different locations (M1–M15) of Uttarakhand state, India.

Site	Age Group	Target Hazard Quotient (THQ)								Health Risk Index (HRI) ^
		Cd	Cr	Cu	Fe	Pb	Mn	Ni	Zn	
M1	Child	0.0033	0.1413	0.0101	0.0014	0.0001	0.0392	0.0098	0.0029	0.2082
	Adult	0.0008	0.0323	0.0021	0.0003	0.0001	0.0090	0.0022	0.0007	0.0474
M2	Child	0.0025	0.1055	0.0075	0.0015	0.0001	0.0310	0.0090	0.0028	0.1599
	Adult	0.0006	0.0241	0.0016	0.0003	0.0001	0.0071	0.0021	0.0006	0.0364
M3	Child	0.0038	0.1113	0.0079	0.0014	0.0001	0.0256	0.0064	0.0026	0.1590
	Adult	0.0009	0.0254	0.0012	0.0003	0.0001	0.0058	0.0015	0.0006	0.0357
M4	Child	0.0029	0.1351	0.0097	0.0013	0.0001	0.0299	0.0040	0.0029	0.1858
	Adult	0.0007	0.0309	0.0016	0.0003	0.0001	0.0068	0.0009	0.0007	0.0419
M5	Child	0.0021	0.1256	0.0090	0.0011	0.0001	0.0248	0.0028	0.0024	0.1677
	Adult	0.0005	0.0287	0.0020	0.0003	0.0001	0.0057	0.0006	0.0005	0.0383
M6	Child	0.0025	0.1324	0.0095	0.0012	0.0001	0.0365	0.0023	0.0026	0.1869
	Adult	0.0006	0.0303	0.0017	0.0003	0.0001	0.0083	0.0005	0.0006	0.0422
M7	Child	0.0017	0.0999	0.0071	0.0012	0.0001	0.0270	0.0039	0.0028	0.1436
	Adult	0.0004	0.0228	0.0018	0.0003	0.0001	0.0062	0.0009	0.0006	0.0329
M8	Child	0.0029	0.1049	0.0075	0.0009	0.0001	0.0237	0.0054	0.0024	0.1478
	Adult	0.0007	0.0240	0.0017	0.0002	0.0001	0.0054	0.0012	0.0005	0.0338
M9	Child	0.0038	0.1022	0.0073	0.0006	0.0001	0.0245	0.0039	0.0025	0.1448
	Adult	0.0009	0.0234	0.0017	0.0001	0.0001	0.0056	0.0009	0.0006	0.0331
M10	Child	0.0038	0.1115	0.0080	0.0008	0.0001	0.0279	0.0077	0.0022	0.1619
	Adult	0.0009	0.0255	0.0014	0.0002	0.0001	0.0064	0.0018	0.0005	0.0366
M11	Child	0.0038	0.0923	0.0066	0.0010	0.0001	0.0317	0.0065	0.0024	0.1444
	Adult	0.0009	0.0211	0.0015	0.0002	0.0001	0.0072	0.0015	0.0006	0.0330
M12	Child	0.0033	0.0710	0.0051	0.0008	0.0001	0.0349	0.0099	0.0024	0.1274
	Adult	0.0008	0.0162	0.0022	0.0002	0.0001	0.0080	0.0023	0.0005	0.0302
M13	Child	0.0021	0.1372	0.0098	0.0013	0.0001	0.0374	0.0079	0.0024	0.1979
	Adult	0.0005	0.0313	0.0020	0.0003	0.0001	0.0085	0.0018	0.0005	0.0450
M14	Child	0.0008	0.1252	0.0089	0.0009	0.0001	0.0254	0.0092	0.0022	0.1727
	Adult	0.0002	0.0286	0.0018	0.0002	0.0001	0.0058	0.0021	0.0005	0.0393
M15	Child	0.0025	0.0934	0.0067	0.0011	0.0001	0.0270	0.0052	0.0025	0.1385
	Adult	0.0006	0.0213	0.0014	0.0003	0.0001	0.0062	0.0012	0.0006	0.0315

^: Indicates potential health risk if the value exceeds > 1.00.

## Data Availability

Not applicable.
